# Isolated Parachute Mitral Valve and Calcified Papillary Muscle in a 70‐Year‐Old Female: Case Report

**DOI:** 10.1155/cric/8871063

**Published:** 2026-06-05

**Authors:** Murad Azamtta, Salma Hajj Qasem, Adil Alsweis, Yunis Daralammouri

**Affiliations:** ^1^ Department of Medicine, Faculty of Medicine and Health Sciences, An-Najah National University, Nablus, State of Palestine, najah.edu; ^2^ Department of Cardiology, An-Najah National University Hospital, Nablus, State of Palestine, najah.edu

**Keywords:** asymptomatic, calcified papillary muscle, congenital heart defect, echocardiography, hypertension, isolated, mitral valve replacement, parachute mitral valve

## Abstract

The parachute mitral valve (PMV) is a rare congenital cardiac defect in which all chordae tendineae are attached to one papillary muscle, giving the valve its parachute‐like appearance. This anatomical defect can result in mitral valve stenosis or regurgitation, which can impede normal blood flow. The diagnosis is often shown by echocardiography, which reveals the valve′s unique features. Papillary muscle calcification is an uncommon echocardiographic condition mainly attributed to advancing age, cardiac ischemia, and degenerative valve disease, and its incidence in relation to PMV is not well established. PMV treatment depends on the severity of the condition, with severe instances frequently necessitating mitral valve repair or replacement. It is estimated that approximately two‐thirds of those with PMV will require surgical treatment. In our instance, a 70‐year‐old patient was diagnosed with isolated PMV and calcified papillary muscle during routine echocardiography as part of high blood pressure evaluation. This case demonstrates the diagnostic and therapeutic difficulties related to PMV, particularly if patients are asymptomatic, and emphasizes the importance of continued monitoring and individualized treatment options.

## 1. Introduction

The mitral valve is a dynamic two‐leaflet valve formed out of the mitral annulus (MA), mitral leaflets, chordae tendineae, and papillary muscles (PMs). For the valve to function effectively, all of its components have to be intact and well‐coordinated. It is thus crucial to maintain this integrity for the normal shape and performance of the left ventricle and its relationship with the valve [1, 2].

Parachute mitral valve (PMV) is a rare congenital heart defect that is frequently associated with Shone′s complex. It occurs in around 0.6% among all congenital heart conditions [3–5]. Improper chordal connection is a common characteristic, commonly resulting in one primary point of attachment, typically placed on the posteromedial PM [6–8]. The chordae tendineae in PMV frequently appear undeveloped, with a short, thick, and sticky look [9]. This results in diminished motion of the valve leaflet and a narrower opening in the mitral valve, which frequently causes mitral stenosis [10]. In contrast, in PMV, mitral valve regurgitation is usually caused by extended chordae prolapsing into the left atrium [11].

A true PMV has one PM by which all chordae tendineae connect. In contrast, a parachute‐like mitral valve contains two PM, with all chordae joining to one and the other remaining underdeveloped [11]. In spite of these variances in anatomy and prognosis, both disorders demonstrate comparable symptoms, treatment, and prognosis. Therefore, numerous researchers view them as belonging to a unified category [6, 12]. PMV, particularly when occurring in isolation, may sometimes persist without symptoms into adulthood and is commonly discovered incidentally during an echocardiogram. Typically, patients do not need medical or surgical intervention in the majority of cases [13]. However, some people with PMV have been reported with specific symptoms, such as shortness of breath, palpitations, chest tightness, atrial fibrillation, and, in rare circumstances, sudden death [14]. The long‐term outcome of PMV is influenced by several circumstances, particularly concurrent cardiac disorders. Severity of mitral valve malfunction, the existence of left ventricular outflow tract obstruction, and surgery at a young age are important risk factors influencing the chance of reoperation and total mortality [15]. Early detection along with appropriate treatment are critical for enhancing outcome and decreasing complications for PMV patients.

Although papillary muscle calcification (PMC) is an infrequent echocardiographic condition, it is usually described in conjunction with coronary artery disease, degenerative valve diseases, and systemic medical conditions such as hypertension and chronic renal disease [16–20]. The prevalence of this condition in inherited cardiac abnormalities like PMV remains unknown. Only a few cases have been reported, making the combination of PMV and PMC a rare occurrence of clinical importance.

We present a case of true PMV associated with PMC in a 70‐year‐old female that was accidentally discovered during a routine transthoracic echocardiography (TTE) to assess for increased blood pressure readings.

## 2. Case Report

In April 2024, a 70‐year‐old woman arrived at An‐Najah National University Hospital for further investigation on her newly discovered hypertension. She has a history of dyslipidemia. Her heart rate was within the usual range at 69 beats per minute, and her overall health seemed normal with no notable findings. She had high blood pressure in both arms and legs, measuring 165/89 mmHg. The results of the examination, which included laboratory tests, an ECG, and a chest X‐ray, were normal.

**Figure 1:**
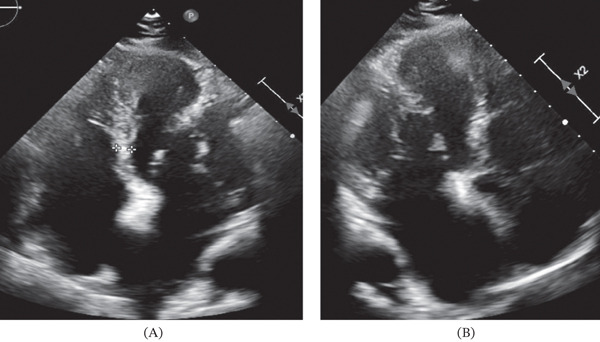
Transthoracic echocardiography—(A) Apical four‐chamber view showing a prominent anterolateral papillary muscle and (B) apical two‐chamber view showing a prominent anterolateral papillary muscle.

A TTE, which was part of the initial evaluation, showed a prominent PM (Figure 1), normal left ventricular size and function, and a dilated left and right atrium. Given the importance of these findings, more investigation was necessary to acquire a thorough assessment of the heart anatomy and function. A transesophageal echocardiography was therefore carried out for a thorough evaluation.

**Figure 2:**
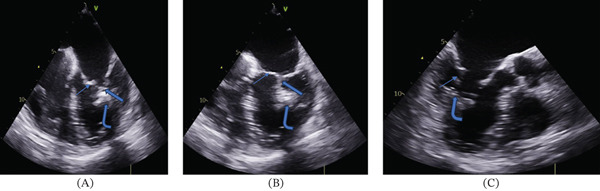
Transesophageal echocardiography apical four chamber (A, B) and three chamber (C) views revealed a calcified anterolateral papillary muscle (curved arrow). The enlarged and extended chordae merged into a single papillary muscle (arrow). Additionally, dysplastic chordae tendineae and thickened mitral valve leaflets (narrow arrow) were observed in the four‐chamber view.

The transesophageal echocardiography revealed a normal‐sized left ventricle with a septal thickness of 12 mm, a dilated left atrium with a diameter of 46 mm and area of 24 cm^2^, and an enlarged right atrium with an area of 19 cm^2^. Systolic function was normal, with an LVEF of 60%. A single calcified PM was seen on the anterolateral side of the left ventricle. The enlarged, calcified and extended chordae merged into a single PM (Figure 2). The mitral valve showed mild regurgitation but no stenosis. There was no evidence of aortic coarctation, MA thickening, or supravalvular mitral membranes.

Therefore, isolated idiopathic parachute mitral valve (IPMV) was the diagnosis.

The patient was given antihypertensive medication and told to monitor her blood pressure for a week. At the time of diagnosis, the patient declined further assessment, including cardiac CT angiography and cardiac catheterization. Despite this, we advised patients to get TTE every 3 to 5 years.

## 3. Discussion

PMV is a rare cardiac abnormality characterized by both mitral valve leaflets attaching to a single PM [6–8]. Diagnosing and treating PMV can be difficult due to its various clinical presentations and concomitant cardiac conditions, necessitating a collaborative approach for optimal patient care. PMV can also be associated with other heart problems such as patent ductus arteriosus, aortic coarctation, ventricular septal defect, and left‐sided obstructive lesions [6, 21].

PMC is a rare condition, recorded in less than 1% of postmortem hearts and more usually associated with ischemic heart disease, hypertension, chronic renal disease, or degenerative valve pathology. Isolated or age‐related PMC has also been observed in the absence of significant cardiac conditions. [16–20]. The occurrence of PMC and congenital mitral anomalies, such as PMV, is uncommon and has only been recorded in isolated case reports. Calcification can further restrict PM and chordal motion, exacerbate valvular dysfunction and challenge surgical replacement or repair.

PMV is also often associated with the Shone complex, a condition first identified over five decades ago. This complex includes several left‐sided heart defects, such as a paravalvular ring in the left atrium (observed in 75%–100% of cases), subaortic stenosis (seen in 50%–85%), aortic coarctation (occurring in 25%–50%), and a parachute‐like mitral valve (present in 25%–50% of cases) [22]. The Shone complex represents a developmental disorder affecting multiple parts of the left heart, creating significant challenges in both diagnosis and treatment due to the variety of associated abnormalities.

Abnormal detachment of the trabecular ridge from the left ventricular wall occurs between the 5th and 19th weeks of gestation, resulting in the creation of a parachute‐like mitral valve and underdeveloped chordae tendineae. True PMV occurs when the posterior and anterior sections of the ridge combine to form a single PM [23]. PMV is distinguished by insufficiently formed chordae tendineae, which causes the tendons to shorten and thicken, limiting the movement of the valve flaps and potentially causing mitral stenosis. Furthermore, mitral regurgitation is commonly caused by leaflet prolapse [11].

Identifying PMV can be difficult, especially prenatally, due to ultrasound′s low efficacy, with the majority of instances being detected after birth. TTE is the primary way of diagnosis, revealing a single PM, parachute‐shaped leaflets, extended chordae doming, and a bigger left atrium [12]. TEE, cardiac MRI, and cardiac CT angiography are advanced imaging modalities that provide detailed assessments of valve structure and function, helping in diagnosis and guiding surgical decisions for symptomatic patients [24].

Regardless of how the patient was identified, the treatment plan for PMV is mostly determined by his or her symptoms and hemodynamic condition. Severe narrowing of the mitral valve is a significant challenge, necessitating surgery in both children and adults. Mitral regurgitation is another condition that affects this group of people. Approximately two‐thirds of PMV patients require surgical surgery for mitral valve issues [15, 25]. Furthermore, surgical intervention is frequently required for other congenital cardiac anomalies, particularly those that cause blockages on the left side of the heart, such as supramitral valve rings, subaortic obstructions, stenotic bicuspid aortic valves, and aortic coarctation [15].

Because of the small annulus size, the need for lifelong anticoagulation, and the increased chance of subsequent reoperations, mitral valve replacement is clinically and technically challenging [15]. Medication for those with this condition primarily relieves the symptoms of heart failure and, if present, arrhythmias [10].

It is critical to undertake regular follow‐ups for an asymptomatic patient diagnosed with valvular heart disease (VHD) to monitor the condition′s progression. The timetable for repeat echocardiograms should be determined by the individual valve lesion, its severity, known progression rates, and the impact on the affected ventricle. Patients should get a history and physical examination at least once a year. Furthermore, any new symptoms or changes seen during physical examinations should prompt a follow‐up TTE to assess how the valve lesion is affecting cardiac function and decide the best course of action [26].

In our case, a 70‐year‐old woman was incidentally found to have isolated PMV with a calcified anterolateral PM during evaluation for hypertension. With the existence of calcification, she had only mild mitral regurgitation and had been asymptomatic. This implies that the combination of PMV and PMC may not always result in severe valvular dysfunction, highlighting the significance of thorough imaging evaluation. Identification of PMC in PMV is clinically important because it can alter prognosis, surgical strategies, and ongoing follow‐up methods. Asymptomatic patients with minor valvular abnormalities can be managed conservatively, with periodic echocardiography advised every 3–5 years [15, 26].

The present case highlights the importance of careful echocardiographic examination in elderly individuals with congenital mitral abnormalities, as well as the uncommon association of PMV with PM calcification. Recognition of this combination has become essential for guiding appropriate treatment for patients and providing timely intervention if valvular function declines.

## 4. Conclusion

We report a rare incidental finding of an isolated PMV associated with PM calcification in a 70‐year‐old patient. This uncommon combination emphasizes the significance of a complete echocardiographic examination in detecting anatomical and degenerative alterations to the mitral apparatus, even in asymptomatic people. Recognizing PM calcification in the setting of congenital abnormalities is clinically important because it can influence valve function, long‐term prognosis, and the complexity of subsequent surgical or interventional procedures. Regular surveillance with specific follow‐up is the cornerstone of such patients′ medical care.

**Nomenclature**
PMV: parachute mitral valve
PMC: papillary muscle calcification
TTE: transthoracic echocardiography
TEE: transesophageal echocardiography
IPVM: isolated parachute mitral valve
CT: computed tomography

## Funding

No funding was received for this manuscript.

## Ethics Statement

In accordance with ethical and legal guidelines, the patient gave written informed consent for the publication of this case.

## Conflicts of Interest

The authors declare no conflicts of interest.

## Data Availability

The data that support the findings of this study are available from the corresponding author upon reasonable request.
